# An Interdisciplinary Approach to Reducing Serious Safety Events by Implementing a Proactive Safety Framework

**DOI:** 10.1097/pq9.0000000000000831

**Published:** 2025-08-29

**Authors:** Caitlin Ehret, Rae-Jean Hemway, Vivien Yap, Priyanka Tiwari, Kiran Gadani-Patel, Megan Toal, Juliana Romano, Lauren Totonis, Emily Branitz-Lenhart, Felicia Alleyne, Brienne Lubor, Christopher Mazzeo, Erin Kelly, Snezana Nena Osorio

**Affiliations:** From the * Quality and Patient Safety, New-York Presbyterian Hospital, New York, N.Y.; † Department of Pediatrics, NewYork-Presbyterian Hospital, New York, N.Y.; ‡ Department of Pediatrics, Weill Cornell Medical Center, New York, N.Y.

## Introduction:

Children’s hospitals across the country have experienced an increase in their serious safety event rate (SSER) over the past 2 years. This is largely attributable to the “pediatric surge” in late 2022/early 2023, which accelerated the staff turnover and attrition that began increasing during the COVID-19 pandemic.^1–4^ At Komansky Children’s Hospital, several root cause analyses were conducted for these events, and each one identified communication among staff as one of the root causes. In July 2023, we began implementing proactive safety huddles across our 4 inpatient units as part of the Solutions for Patient Safety Proactive Safety Framework initiative, with the primary aim of decreasing our SSER (number of serious safety events/10,000 patient days) by 50% by December 31, 2024.

## Methods:

**Fig. 1.**
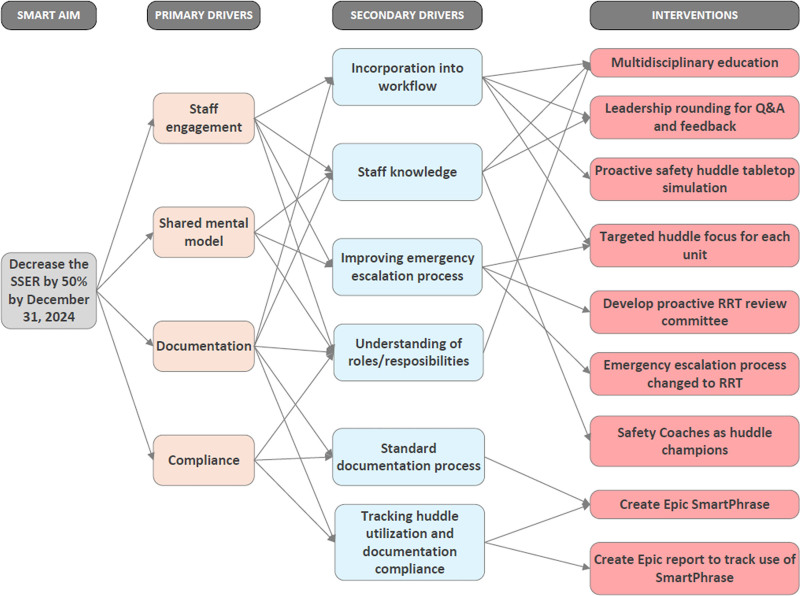
Key driver diagram identifying the primary and secondary drivers that were used to generate tertiary drivers. The tertiary drivers were used to define the sequential interventions that were implemented. RRT, rapid response team; Q&A, question and answer session.

This ongoing QI study used the Model for Improvement with a series of sequential interventions. Our interdisciplinary QI team consisting of nurse champions, physicians, unit nursing and physician leaders, advanced practice providers, and a quality and patient safety specialist created a key driver diagram (Fig. 1). We used the following family of measures: the SSER (outcome), the number of proactive safety huddles (process) and compliance with completing monthly hospital acquired infection audits (balancing). We tested the following interventions through 6 plan–do–study–act cycles including staff education about the proactive safety framework, implementation of the huddles, rounding on units to gather frontline staff input to help identify areas of focus for each unit, targeting huddles to specific patient populations on each unit, developing an Epic SmartPhrase for documenting huddles, and engaging our safety coaches as champions. We also launched an interdisciplinary committee to proactively review all rapid response activations for opportunities to improve our processes before the occurrence of a safety event. We collected data via chart audits, Epic reports, and our hospital event reporting system. Data were analyzed using the statistical process control “U” chart. We applied Associates for Process Improvement rules to detect special cause variation.

## Results:

**Fig. 2.**
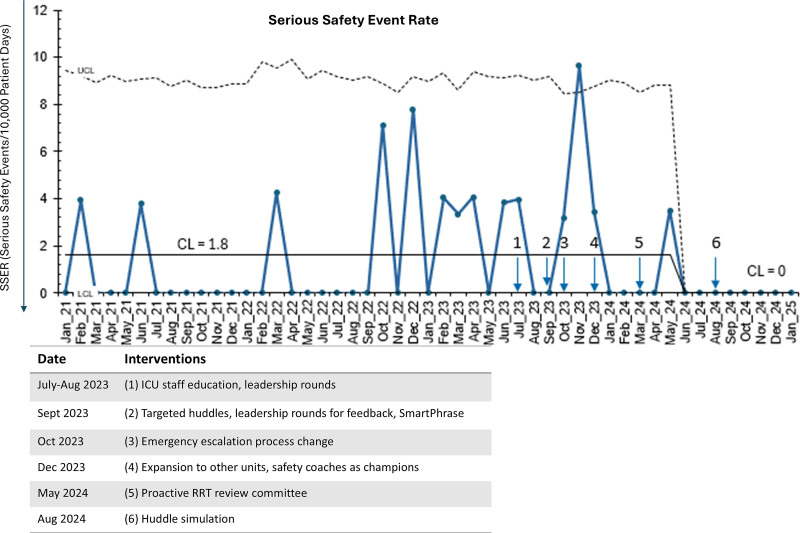
U chart showing the SSER from January 2021 to January 2025. January 2021–June 2023 is baseline data, with the sequential interventions beginning in July 2023. Timing of each sequential plan–do–study–act cycle annotated numerically on the chart, with corresponding descriptions in the accompanying table. A centerline shift from 1.8 serious safety events per 10,000 patient days to 0 SSEs per 10,000 patient days beginning in June 2024 is shown. Directional arrow along *y*-axis indicates desired direction of the SSER. CL, centerline; ICU, intensive care unit; RRT, rapid response team.

We reduced the SSER from 1.6 to 0/10,000 patient days (Fig. 2). There was an average of 7 huddles documented monthly. There was no decrease in the rate of compliance in performing monthly hospital acquired infection audits. Our hospital-wide process for performing these audits changed in October 2024, leading to an increase from 28 audits per month on average to 47 audits per month. An unanticipated result was an increase in enrollment in our safety coach program, with 8 new coaches enrolled since August 2023, compared with 1 new enrollment before rollout.

## Conclusions:

Adoption of a proactive safety framework with implementation of safety huddles can improve interdisciplinary communication and may lead to a reduction in the SSER. Future interventions include expanding the proactive safety huddles to other clinical situations, formalizing the documentation process in our electronic medical record system, and expanding to other inpatient pediatric areas within our hospital system.
